# The Effects of Pre-conditioning on Exercise-Induced Muscle Damage: A Systematic Review and Meta-analysis

**DOI:** 10.1007/s40279-023-01839-8

**Published:** 2023-05-09

**Authors:** Lachlan Boyd, Glen B. Deakin, Baily Devantier-Thomas, Utkarsh Singh, Kenji Doma

**Affiliations:** College of Healthcare Sciences, Sports and Exercise Science, James Cook University, 1 James Cook Drive, Rehabilitation Sciences Building, Douglas, Townsville, QLD 481 Australia

## Abstract

**Background:**

Several studies have utilised isometric, eccentric and downhill walking pre-conditioning as a strategy for alleviating the signs and symptoms of exercise-induced muscle damage (EIMD) following a bout of damaging physical activity.

**Objectives:**

This systematic review and meta-analysis examined the effects of pre-conditioning strategies on indices of muscle damage and physical performance measures following a second bout of strenuous physical activity.

**Data Sources:**

PubMed, CINAHL and Scopus.

**Eligibility Criteria:**

Studies meeting the PICO (population, intervention/exposure, comparison, and outcome) criteria were included in this review: (1) general population or “untrained” participants with no contraindications affecting physical performance; (2) studies with a parallel design to examine the prevention and severity of muscle-damaging contractions; (3) outcome measures were compared using baseline and post-intervention measures; and (4) outcome measures included any markers of indirect muscle damage and muscular contractility measures.

**Participants:**

Individuals with no resistance training experiences in the previous 6 or more months.

**Interventions:**

A single bout of pre-conditioning exercises consisting of eccentric or isometric contractions performed a minimum of 24 h prior to a bout of damaging physical activity were compared to control interventions that did not perform pre-conditioning prior to damaging physical activity.

**Study Appraisal:**

Kmet appraisal system.

**Synthesis Methods:**

Quantitative analysis was conducted using forest plots to examine standardised mean differences (SMD, i.e. effect size), test statistics for statistical significance (i.e. Z-values) and between-study heterogeneity by inspecting I^2^.

**Results:**

Following abstract and full-text screening, 23 articles were included in this paper. Based on the meta-analysis, the pre-conditioning group exhibited lower levels of creatine kinase at 24 h (SMD =  − 1.64; Z = 8.39; *p* = 0.00001), 48 h (SMD =  − 2.65; Z = 7.78; *p* = 0.00001), 72 h (SMD =  − 2.39; Z = 5.71; *p* = 0.00001) and 96 h post-exercise (SMD =  − 3.52; Z = 7.39; *p* = 0.00001) than the control group. Delayed-onset muscle soreness was also lower for the pre-conditioning group at 24 h (SMD =  − 1.89; Z = 6.17; *p* = 0.00001), 48 h (SMD =  − 2.50; Z = 7.99; *p* = 0.00001), 72 h (SMD =  − 2.73; Z = 7.86; *p* = 0.00001) and 96 h post-exercise (SMD =  − 3.30; Z = 8.47; *p* = 0.00001). Maximal voluntary contraction force was maintained and returned to normal sooner in the pre-conditioning group than in the control group, 24 h (SMD = 1.46; Z = 5.49; *p* = 0.00001), 48 h (SMD = 1.59; Z = 6.04; *p* = 0.00001), 72 h (SMD = 2.02; Z = 6.09; *p* = 0.00001) and 96 h post-exercise (SMD = 2.16; Z = 5.69; *p* = 0.00001). Range of motion was better maintained by the pre-conditioning group compared with the control group at 24 h (SMD = 1.48; Z = 4.30; *p* = 0.00001), 48 h (SMD = 2.20; Z = 5.64; *p* = 0.00001), 72 h (SMD = 2.66; Z = 5.42; *p* = 0.00001) and 96 h post-exercise (SMD = 2.5; Z = 5.46; *p* = 0.00001). Based on qualitative analyses, pre-conditioning activities were more effective when performed at 2–4 days before the muscle-damaging protocol compared with immediately prior to the muscle-damaging protocol, or 1–3 weeks prior to the muscle-damaging protocol. Furthermore, pre-conditioning activities performed using eccentric contractions over isometric contractions, with higher volumes, greater intensity and more lengthened muscle contractions provided greater protection from EIMD.

**Limitations:**

Several outcome measures showed high inter-study heterogeneity. The inability to account for differences in durations between pre-conditioning and the second bout of damaging physical activity was also limiting.

**Conclusions:**

Pre-conditioning significantly reduced the severity of creatine kinase release, delayed-onset muscle soreness, loss of maximal voluntary contraction force and the range of motion decrease. Pre-conditioning may prevent severe EIMD and accelerate recovery of muscle force generation capacity.

**Supplementary Information:**

The online version contains supplementary material available at 10.1007/s40279-023-01839-8.

## Key Points

**Table:** 

Exercise-induced muscle damage (EIMD) decreases the functional capacity of untrained individuals, following strenuous physical activity.
Performing pre-conditioning activities several days to weeks prior to strenuous physical activity reduces the severity of EIMD and decreases recovery time.
Pre-conditioning activities appear to be most effective if performed 2–4 days prior to muscle-damaging exercises, and with greater volume and intensity.
Although greater volume and intensity of pre-conditioning activities may cause EIMD, the level of EIMD is notably lower than the initial exposure to EIMD exercises, indicating that pre-conditioning activities may be effective in reducing the level of EIMD following initial exposure to unfamiliar strenuous exercises.

## Introduction

Exercise-induced muscle damage (EIMD) is a phenomenon that typically occurs after a bout of intense physical exertion, with eccentric muscle contractions causing the highest degree of damage because of forced lengthening during the cross-bridge cycle [1]. Exercise-induced muscle damage normally occurs within 48 h and can last between 2 and 10 days. Symptoms include localised muscle soreness (delayed-onset muscle soreness, [DOMS]) and swelling, increased blood biomarkers caused by muscle cell damage (e.g. creatine kinase [CK]), decreased voluntary force production and an increase in the perceived difficulty of movement [2–4]. These symptoms result in detrimental effects during subsequent training sessions, such as reductions in force and power output, impaired proprioception and movement compensation, and decreased training effort, thus impairing the quality of training and increasing the potential for injury in athletes [5, 6].

Once EIMD occurs because of strenuous exercise, a subsequent bout of an equivalent activity will result in less severe EIMD, known as the repeated bout effect (RBE) [7–9]. Musculoskeletal structures exposed to a previously damaging stimulus are protected against a subsequent large EIMD response due to neural and structural adaptation, including improved motor unit recruitment and a rate of force production increase [8]. Hence, once an acute adaptation occurs, the musculoskeletal structures exhibit accelerated recovery dynamics following a strenuous bout of exercise. Various studies have observed the duration of protection against EIMD, with the level of protection being maintained for 2–8 weeks after the first bout of eccentric exercise [10–12], and approximately 6 months until return to conditions prior to the muscle-damaging exposure [9].

The traditional form of RBE would require greater recovery following the initial bout of exercise, given the initial exposure to high levels of physiological stress and the undesirable signs and symptoms of EIMD before undertaking subsequent training bouts. To circumvent these training difficulties, studies have attempted to attenuate EIMD using lesser damaging to non-damaging pre-conditioning activities. Several types of pre-conditioning activities have been examined, including: low-intensity and volume eccentric contractions that range from 10 to 40% of maximum contraction effort [13, 14]; short duration (3–5 s) maximal voluntary contractions (MVCs) repeated several times [10, 15] and short duration downhill walking (5 min) utilising the eccentric nature of a downhill gait to attenuate damage induced by the long duration of downhill walking a week later [16]. These pre-conditioning interventions produced no measurable change in indirect biomarkers of EIMD (e.g. CK), and did not produce DOMS, inflammation or an extended reduction in force production [17–19]. Furthermore, participants exposed to various pre-conditioning activities demonstrated significantly lower CK and DOMS values, whilst force production measures recovered sooner after muscle-damaging exercises, when compared with control groups. However, pre-conditioning strategies also have a short duration of effectiveness and are required to be performed 1 day to 2 weeks prior to any muscle-damaging activity, with 24–48 h as the preferred time period for isometric contractions [15, 17, 20] and weeks for non-damaging eccentric muscle contractions [21–23]. Whilst not fully understood, it is believed that pre-conditioning activities exhibit protection against EIMD using similar mechanisms to that of traditional RBE, such as neurological priming, extra-cellular matrix remodelling and pennation angle changes to provide a short-term adaptation, allowing greater transmission of force and improved joint congruency while maintaining neural excitation [4, 24–26]. More recent evidence also indicates a smaller displacement of the myotendinous junction as a potential of the pre-conditioning effect on muscle damage [27]. Therefore, the pre-conditioning effect and RBE both exhibit protection from EIMD following the subsequent muscle-damaging bouts and are underpinned by similar mechanisms. However, with the pre-conditioning effect, the exercises during the first bout are deliberately selected to lower the level of EIMD (e.g. lower intensity, lower volume, shorter muscle length) in preparation for a more intense second bout of muscle-damaging exercises. Conversely, the RBE phenomenon is usually considered when the muscle-damaging exercises are prescribed similarly, if not identically, between the first and subsequent bouts with the intention to cause a high level of EIMD following the first bout.

Given the ability to induce an RBE with minimal EIMD symptomatology, pre-conditioning activities may be a useful tool for rehabilitation or concurrent training scenarios where high levels of EIMD are expected, although are not desirable. For example, attenuating EIMD for athletes returning to resistance training after a long hiatus because of an injury may decrease the recovery time needed between rehabilitation sessions, and thereby speed up the return to play time [14]. Individuals considering introducing resistance exercises as part of their normal training programme, such as endurance athletes or youth athletes, may also consider pre-conditioning activities to minimise the level of EIMD during the initial period of a resistance training programme.

There is clear evidence to date regarding the effectiveness implementing pre-conditioning activities prior to muscle-damaging protocols to ameliorate the level of EIMD. However, the methodologies used to implement pre-conditioning thus far have varied. For example, including short- and long-duration MVC appears to have a potent prophylactic effect against EIMD when used prior to high-intensity eccentric contractions [10, 17]. Low-intensity eccentric contractions, low-volume eccentric contractions and downhill walking have been performed on various muscle groups, attenuating a second more intense bout of eccentric contractions without first causing EIMD [20, 23, 28]. Because of the variety of contraction times, intensities and volumes used for pre-conditioning all reducing EIMD with some degree of effectiveness, it is difficult for the practitioner to discern best pre-conditioning practice. A recent review examining the effect of isometric pre-conditioning highlighted that isometric pre-conditioning attenuates EIMD in populations with little or no prior eccentric contraction exposure. However, the review highlighted that pre-conditioning has not yet been explored in a trained population and may not be effective, owing to the long-term prophylactic effects of eccentric contractions [29]. This review also postulated that the potency of attenuation of EIMD may be related to the number of maximal isometric contractions. However, to date, no appraisal of the quality of studies examining isometric or EPC has been completed, nor a meta-analysis conducted based on pooled data of multiple studies. Therefore, a systematic review and meta-analysis are warranted to provide insight into the effectiveness of these different pre-conditioning strategies. Consequently, the aim of the current systematic review and meta-analysis is to evaluate the effectiveness of pre-conditioning strategies in preventing EIMD, give some insight to practitioners regarding implementation and to highlight areas of further research.

## Methods

**Fig. 1:**
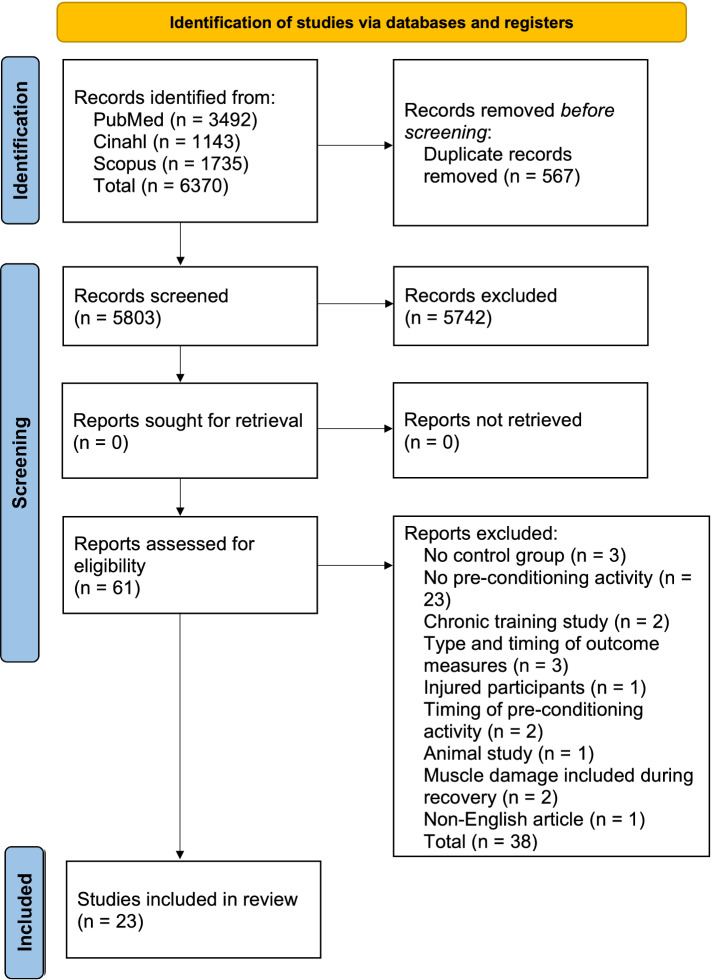
Selection process detailing the search procedure for assessing eligibility for inclusion in a systematic review and meta-analysis

This systematic review and meta-analysis used the PRISMA (Preferred Reporting Items for Systematic Reviews and Meta-Analyses) guidelines [30] for the methodology and reporting of data (Fig. 1). A PICO (population, intervention/exposure, comparison and outcome) approach was followed to determine study eligibility and inclusion in this review.

### Inclusion/Exclusion Criteria

Studies meeting the following PICO criteria were included in this review.

1. *Population* healthy adults.
2. *Intervention* studies with a parallel (between-group) design to examine the prevention and severity of muscle-damaging contractions following a bout of pre-condition exercises (e.g. a single bout of 10% pre-conditioning eccentric contractions prior to a single bout of 50 maximal eccentric contractions in the pre-conditioning group compared with a control group that performed a single bout of 50 maximal eccentric contractions without pre-conditioning activities).
3. *Comparison* outcome measures were compared between groups based on measures at 24 h, 48 h, 72 h and 96 h after the muscle-damaging protocol.
4. *Outcome* outcome measures included common markers of indirect muscle damage (e.g. CK, DOMS, joint range of motion [ROM]) and muscular contractility measures (e.g. isometric force and isokinetic torque).

Studies were excluded if: (1) they were reported in a language other than English with no translation; (2) they were clinical in nature (i.e. participants were recovering from a trauma such as a stroke or injury); (3) no clear measures of EIMD were reported; (4) no comparative groups were presented; and (5) they were reported as abstracts, reviews or case studies.

### Search Strategy

The following literature search was performed on 7 April, 2022 across three electronic databases (PubMed, CINAHL and Scopus). For the PubMed search, mesh terms were employed with separate strings, including: (1) Humans; (2) Muscle-damage protocols (Exercise or Exercise therapy/methods or Physical conditioning, human/adverse effects or Physical conditioning, human/methods or Exercise test or Physical exertion/physiology or Resistance training/methods or Resistance training/adverse effects); and (3) muscle damage markers (Creatine kinase or Myoglobin or L-Lactate Dehydrogenase or Interleukin-6 or Interleukin-8 or Interleukin-1 or C-Reactive Protein"[Mesh] or Tumour Necrosis Factor-alpha"[Mesh] or Inflammation or Oxidative stress or Pain/etiology or Myalgia or Musculoskeletal pain and Muscle, skeletal or Quadriceps muscle or" or Arm or Elbow joint or Knee joint). A free text search was also conducted in PubMed to source papers that were currently in press, including: (Muscle Damage or Creatine Kinase or Myoglobin or Lactate Dehydrogenase or Muscle Soreness, or Interleukin 6 or Interleukin 8 or C-reactive Protein or Tumour Necrosis Factor or TNF or Oxidative Stress) and (Eccentric or Concentric or Isometric or Pre-conditioning or Priming) and (Repeated Bout or Second Bout or Subsequent Bout or Alleviat* or Recover* or Adapt* or Adaptation). An equivalent free text search was used for the Cinahl and Scopus databases. Furthermore, reference lists from included studies were screened as a supplementary search.

### Selection Process

A list of abstracts extracted from the literature search was screened for eligibility by two authors with sport and exercise science backgrounds (LB and BDT). Abstracts were classified as either meeting the inclusion criteria (yes) or not meeting the inclusion criteria (no). Once screening was completed, the full texts of all included abstracts were further screened using the same inclusion criteria. Any discrepancies for the screening of the abstracts and the full-text search between the two authors involved a third author (KD), until a consensus was reached.

### Data Extraction, Quality Assessment and Risk of Bias

Upon completion of the abstract and full-text screening, information relating to the study design, number of participants, study aims and main findings was extracted. The mean ± standard deviation of all outcome measures following the muscle-damage protocols at 24 h, 48 h, 72 h and 96 h post-exercise for the pre-conditioning and control groups were entered into a custom-built Excel sheet. For studies where outcome measures were reported as figures, an e-mail requesting raw data was sent to corresponding authors. When a response was not provided by the corresponding authors, data from figures were extracted using a digitising software (ImageJ; National Institutes of Health, Bethesda, MD, USA). The methodological quality of each study was assessed using the Kmet appraisal checklist for quantitative studies [31]. The Kmet scoring tool consists of a three-point ordinal scoring system (‘yes’ = 2, ‘partial’ = 1, ‘no’ = 0), with appropriate validity and reproducibility [31]. Given that several of the Kmet criteria were inapplicable for the distinct study designs employed by the included studies, these criteria were either modified or replaced, with potential confounding variables common within the study design of EIMD studies. Specifically, the scores for criterion 4 (subject characteristics or input variables/information sufficiently described, additional scoring considerations for section) were modified to the following: a score of 2 if participants were clearly described as ‘untrained’ for more than 6 months and criteria for inclusion regarding training history were clearly outlined; a score of 1 if participants were described as ‘untrained’ but criteria for inclusion regarding training history were not clearly outlined; and a score of 0 if the participant’s training history was not clearly defined or participants were trained with no cessation period prior to study commencement. Criterion 5 (if random allocation to a treatment group was possible, is it described?) was modified to the following: a score of 2 if participants were matched by MVC or another appropriate baseline measure and randomised with the description of the method used; a score of 1 if participants were matched by MVC or other criteria but not randomised, or a method of randomisation was not mentioned; a score of 0 if participant allocation to a group was not clearly described. Criterion 6 (interventional and blinding of investigators to intervention was possible, is it reported?) was modified to the following: if baseline or pre-test measures included eccentric or isometric contractions, was a washout period observed prior to treatment commencing? A score of 2 if adequate time was allocated to allow for the cessation of any RBE occurring from the contraction present in the study; a score of 1 if concentric contractions were measured only, some time may be present between testing and treatment; a score of 0 if eccentric or isometric contractions were present and time allocated between baseline or pre-test measures not adequate to extinguish a repeated bout effect. Criterion 7 (If interventional and blinding of subjects to intervention was possible is it reported?) was modified to the following: were measures of dependent variables taken at the same time of day or within a specified window? A score of 2 if the time of day that dependent variables were measured was specified in the methodology (or elsewhere in the study); a score of 1 if the time of day was not specified but a similar time of day could be assumed based on evidence elsewhere, for example, figures or tables; a score of 0 if the time of day was not specified in the methodology and cannot be assumed based on other information present in study. Criteria 12. (Controlled for confounding?) were modified to: were dependent variables recorded at appropriate intervals? A score of 2 if dependent variables were measured daily with no less than 2 consecutive days post-intervention; a score of 1 if dependent variables were measured at regular intervals but may skip some 24-h periods; a score of 0 if dependent variables were measured randomly with no reasoning behind the schedule. Individual scores were reported for each study using an equation [31], and overall scores of < 50, 50–66, 67–82 and > 82 were expressed as ‘poor’, ‘fair’, ‘good’ and ‘excellent’ [32], respectively. The certainty of evidence was also assessed using the Grading of Recommendations Assessment, Development and Evaluation (GRADE).

### Certainty of Evidence

The certainty of evidence for each outcome was examined by two authors (KD and US) using the Grading of Recommendations Assessment, Development and Evaluation (GRADE) protocol with ratings that ranged from very low to high levels of certainty [33–35]. The evidence was downgraded based on the following reasons: (i) risk of bias in studies: downgraded if the average Kmet scores were fair (< 67%) or by two levels if they were poor (< 50%); (ii) indirectness: a low risk of indirectness was caused because of the specificity of populations, interventions, comparators and outcomes being guaranteed by the eligibility criteria; (iii) a risk of publication bias: the judgements were downgraded by one level if there was a publication bias; (iv) inconsistency: downgraded by one level if the interstudy heterogeneity (*I*^2^) was high (> 75%); and (v) imprecision: downgraded by one level when < 800 participants were available [36].

### Statistical Analysis

A meta-analysis was conducted to determine the effectiveness of pre-conditioning strategies for EIMD prevention using the RevMan (version 5.4) statistical software (2020; The Nordic Cochrane Centre, Copenhagen, Denmark). All outcome measures of interest relating to EIMD (CK, DOMS, ROM and muscular contractility) from each study were reported as mean ± standard deviation. The outcome measures were compared between the control group (no pre-conditioning) and pre-conditioning group at 24 h, 48 h, 72 h and 96 h after the muscle-damaging protocol. For studies where a control group was not included, the group that performed equivalent exercises between pre-conditioning (i.e. first bout) and the subsequent muscle-damaging protocol (i.e. second bout) was treated as the control group. For example, in the study by Nosaka et al. [18], participants were separated into three separate groups that performed two maximal eccentric contractions (2ECC), six maximal eccentric contractions (6ECC) or 24 maximal eccentric contractions (24ECC) as pre-conditioning exercises (i.e. first bout). All the participants then performed 24 maximal eccentric contractions 2 weeks later as their muscle-damaging protocol (i.e. second bout). In this instance, we extracted data following the pre-conditioning activity (i.e. first bout) for the 24ECC group and treated these data as our control group. The data extracted following the muscle-damaging exercises (i.e. bout 2) from 2 and 6ECC were treated as the pre-conditioning groups in our meta-analysis. Once all data were extracted from each study, forest plots were generated to determine differences between groups using the random-effects model. The magnitude of differences (i.e. pre-conditioning vs control groups) was calculated based on standardised mean differences, with values of 0.2, 0.5 and 0.8 considered small, medium and large, respectively. The between-group differences based on the pooled data were quantified as *Z*-values from the forest plot, in conjunction with *p*-values to determine the level of statistical significance, and the alpha level set at 0.05. Thus, effectiveness of the pre-conditioning activities was ascertained according to standardised mean differences and statistical significance between the pre-conditioning and control groups. The heterogeneity of the data points was assessed using *I*^*2*^ statistics, with values of 25%, 50% and 75% classified as low, moderate and high, respectively. To determine a potential publication bias, Egger’s test was conducted using the Statistical Package of Social Sciences (SPSS, version 26; IBM, Corporation, Armonk, NY, USA).

## Results

### Systematic Literature Search

A total of 5803 abstracts were extracted and screened according to the inclusion criteria from PubMed, Scopus and CINAHL databases. Following screening, 5742 abstracts were excluded, and the remaining 61 full-text articles were further assessed, leaving 23 articles for inclusion (Fig. 1).

### Outcome Measures

Primary outcome measures included in the current review were common measures associated with EIMD; biochemical markers, physical performance, post-exercise muscle soreness, swelling and degrees of joint motion. These measures have previously been used as strong indicators of EIMD. Biochemical markers of indirect muscle damage included CK and myoglobin, whilst physical performance measures included MVC, maximal isometric torque, peak isometric force, peak eccentric force, peak concentric force and maximal eccentric contraction. Post-exercise muscle soreness was classified based on a subjective rating of muscle soreness using visual analogue scales. Swelling was measured externally by measuring the girth circumference in millimetres of the affected limb segment. Degrees of joint motion were taken as the percentage of change in degrees of the affected limb, or a true change in degrees, symptomatic of reduced contractility and tissue swelling.

### Participants

Studies that included all groups with pre-conditioning activities had a total sample size of 427 participants (Table 1). The average age, height and body mass (including range) for isometric pre-conditioning and ECC pre-conditioning groups were 20.3 years (21–70 years), 164 cm (159–178 cm) and 62.4 kg (58–89 kg), respectively. Similar characteristics were present for studies with those that had a control group (i.e. no pre-conditioning activity), for which the total sample size was 261 participants with an average age, height and body mass (including range) of 22.1 years (20–30 years), 160 cm (159–187 cm) and 71.4 kg (50–91 kg). When baseline measures were compared between the isometric pre-conditioning and ECC pre-conditioning groups across both study designs, no significant differences were identified (*p* > 0.05), indicating between-group measures were relatively homogenous.

**Table 1:** Participant characteristics and methodological quality of included studies

Study (first author)	Sample size (*n*)	Physical characteristics	Comparative groups	Inclusion	Overall quality
Barreto [17]	ISO + ECC-S group: 16 ISO + ECC-F group: 16 ECC S group: 16 ECC-F group: 16	ISO + ECC-S, ISO + ECC-F, ECC-S and ECC-F groups: Age 21.7 ± 3.1 years Height 174 ± 5.0 cm Mass 74.7 ± 13.6 kg	Experimental group: ISO + ECC-S and ISO + ECC-F Control group: ECC- S and ECC-F	Untrained young men who had not performed regular resistance training in the previous 6 months	Excellent
Brown [28]	ECC 10 group: 7 ECC 30 group: 9 ECC 50 group: 8	ECC 10 group: Age 20.5 ± 1.1 years Mass 68.3 ± 9.3 kg ECC 30 group: Age 20.1 ± 0.8 years Mass 63.6 ± 13.9 kg ECC 50 group: Age 22.1 ± 2.4 years Mass 69.3 ± 11.5 kg	Experimental group: ECC 10 and ECC 30 following the second bout after the muscle-damaging protocol Control group: ECC 50 following the first bout before the muscle-damaging protocol	Untrained participants	Good
Burt [37]	Low–high groups: 8 High-high group: 8	Low–high group: Age 26 ± 5 years Height 179 ± 5.0 cm Mass 79.5 ± 7.8 kg High-high group: Age 27 ± 4 years Height 177 ± 10 cm Mass 76.7 ± 9.8 kg	Experimental group: Low–high following the second bout after the muscle-damaging protocol Control group: High-high following the first bout before the muscle-damaging protocol	Healthy men who regularly participated in endurance exercise (2–3 times weekly) but did not perform lower body resistance training in the previous 6 months	Excellent
Chen [21]	2-day group:13 7-day group: 13 14-day group:13 21-day group: 13 No pre-conditioning group: 13	2-day, 7-day, 14-day, 21-day and no pre-conditioning groups: Age 21.3 ± 1.6 years Height 172.3 ± 4.9 cm Mass 71.4 ± 9.9 kg Body fat 25.1 ± 3%	Experimental group: 2-day group, 7-day group, 14-day group, 21-day group Control group: No pre-conditioning	Sedentary men who had not performed regular resistance, aerobic or flexibility training in the previous year and did not carry heavy objects	Excellent
Chen [2]	ECC 100% group: 13 ECC 80% group: 13 ECC 60% group: 13 ECC 40% group: 13	ECC 100%, ECC 80%, ECC 60% and ECC 40% groups: Age 20.7 ± 1.9 years Height 173.8 ± 5.0 cm Mass 67.6 ± 7.2 kg	Experimental group: ECC 80%, ECC 60% and ECC 40% following the second bout after the muscle-damaging protocol Control group: ECC 100% following the first bout before the muscle-damaging protocol	Physically active men who did not perform regular resistance training	Good
Chen [38]	Maximum ECC group:13 10% ECC group: 13 20% ECC group:13 90 ISO group: 13 20 ISO group: 13	Maximum ECC, 10% ECC, 20% ECC, 90 ISO and 20 ISO groups: Age 20.9 ± 1.8 years Height 174 ± 5.3 cm Mass 67.3 ± 10.6 kg	Experimental group: 10% ECC, 20% ECC group, 90 ISO and 20 ISO following the second bout after the muscle-damaging protocol Control group: Maximum ECC following the first bout before the muscle-damaging protocol	Young men not involved in regular resistance, aerobic or flexibility training in the previous 1 year	Good
Chen [13]	Maximum ECC: 13 Low ECC: 13	Maximum ECC and low ECC groups: Age 66.4 ± 4.6 years Height 164.7 ± 5.4 cm Mass 68.8 ± 7.5 kg Body fat 25.1 ± 3% BMI 25.4 ± 2.2	Experimental group: Low ECC following the second bout after the muscle-damaging protocol Control group: Max ECC following the first bout before the muscle-damaging protocol	Healthy untrained men over 60 years of age	Excellent
Chen [10]	2 MVC-ISO group: 13 10 MVC-ISO group: 13 No pre-conditioning group: 13	2 MVC-ISO, 10 MVC-ISO and no pre-conditioning groups: Age 22.5 ± 1.7 years Height 172.9 ± 5.7 cm Mass 71.7 ± 9 kg	Experimental group: 2 MVC-ISO and 10 MVC-ISO Control group: No pre-conditioning	Young men not engaged in regular resistance, aerobic or flexibility training in the previous year, who did not carry heavy objects regularly	Excellent
Chen [15]	0-day group: 13 2-day group: 13 4-day group:13 7-day group: 13 No pre-conditioning group: 13	Height 174 ± 5.3 cm	Experimental group: 0-day, 2-day, 4-day and 7-day Control group: No pre-conditioning	Young men not engaged in regular resistance, aerobic or flexibility training in the previous year, who did not carry heavy objects regularly	Excellent
Eston [3]	100 ECC group: 5 No pre-conditioning group: 5	Mass 67.3 ± 10.6 kg	Experimental group: 100ECC Control group: No pre-conditioning	Healthy men not accustomed to isokinetic eccentric dynamometry or downhill running	Good
Howatson [39]	ECC 10 group: 8 ECC 45 group: 8	ECC 10 and ECC 45 groups: Age 27 ± 5 years Height 180.6 ± 6.9 cm Mass 83.9 ± 10.5 kg	Experimental group: ECC 10 following the second bout after the muscle-damaging protocol Control group: ECC 45 following the first bout before the muscle-damaging protocol	Participants familiar with resistance training but not accustomed to eccentrically based exercise or isokinetic dynamometry	Good
Huang [22]	10% ECC group: 12 No pre-conditioning group: 12	10% ECC and no pre-conditioning groups: Age 21.6 ± 1.8 years Height 172.4 ± 5.9 cm Mass 68.8 ± 11.8 kg	Experimental group: 10% ECC Control group: No pre-conditioning	Untrained healthy university students	Good
Paddon-Jones [40]	ECC group: 7 Low volume + ECC group: 8 No pre-conditioning group: 8	ECC group, low volume + ECC and no pre-conditioning groups: Age 23.9 ± 4.9 years Height 172.6 ± 8.2 cm Mass 67.4 ± 11.6 kg	Experimental group: ECC group and low volume + ECC Control group: No pre-conditioning	Non-resistance-trained individuals	Good
Lavender [14]	ECC 10%: 9 No pre-conditioning group: 9	ECC 10% and no pre-conditioning groups: Age 21.4 ± 2.6 years Height 171.2 ± 5.4 cm Mass 63.7 ± 8.8 kg Body fat 15.8 ± 4.7%	Experimental group: ECC 10% Control group: No pre-conditioning	Untrained men not involved in resistance training for the previous year	Good
Lima [20]	Isometric pre-conditioning: 15 No pre-conditioning: 15	Isometric pre-conditioning and no pre-conditioning group: Age 22.8 ± 2.3 years Height 1.77 ± 0.04 cm Mass 78.6 ± 8.9 kg	Experimental group: Isometric pre-conditioning Control group: No pre-conditioning	Young men with no strength or endurance training in the previous 6 months	Good
Lin [23]	2-day group: 13 1-week group: 13 2-week group: 13 3-week groups: 13 No pre-conditioning group: 13	2-day, 1-week, 2-week and 3-week and no pre-conditioning groups: Age 21.4 ± 1.8 years Height 172.0 ± 5.5 cm Mass 66.3 ± 6.8 kg	Experimental group: 2 days, 1 week, 2 weeks and 3 weeks Control group: No pre-conditioning	Untrained young men not involved in aerobic, resistance or flexibility training in the previous year	Good
Maeo [7]	Pre-1 week: 14 Pre-4 weeks: 14 No pre-conditioning: 14	Pre-1 week, pre-4 weeks and no pre-conditioning group: Age 20.8 ± 1.4 years Height 165.2 ± 6.5 cm Mass 60.2 ± 6.1 kg	Experimental group: Pre-1 week Pre-4 weeks Control group: No pre-conditioning	Untrained men not involved in aerobic, resistance or flexibility training and had trekking experience	Excellent
Maeo [16]	DW group: 12 LW group: 12 No pre-conditioning group: 12	DW group, LW group and no pre-conditioning groups: Age 23.5 ± 4.5 years Height 1.68 ± 0.07 cm Mass 64.7 ± 6.4 kg	Experimental group: PRE-DW and PRE-LW Control group: No pre-conditioning	Untrained young men not involved in aerobic, resistance or flexibility training and had trekking experience	Good
Miyama Nosaka [41]	Experimental: 8 Control: 8	Age 21.1 ± 1.1 years Height 174.0 ± 7.2 cm, Mass 65.1 ± 8.0 kg	Experimental group: Performed 5 sets of 10 DJs in the second bout Control: Performed 5 sets of 10 DJs in the first bout	Men with little or no experience in resistance training	Good
Nosaka [8]	CON-ECC group: 9 ECC group: 9	CON-ECC and ECC: Age 23.3 ± 6.7 years Height 159 ± 6 Mass 51.4 ± 5.9 kg	Experimental group: CON-ECC Control group: ECC	Women with no experience in resistance training	Good
Nosaka [18]	2 ECC group: 12 6 ECC group: 10 24 ECC group 12	2 ECC group, 6 ECC group, 24 ECC group: Age 20.6 (10–27) years Height 172.7 (159–186) cm Mass 61.6 (50–76.3) kg Body fat 15.9% (9.2–26.1%)	Experimental group: 2 ECC and 6 ECC following the second bout after the muscle-damaging protocol Control group: 24 ECC following the first bout before the muscle-damaging protocol	Non-athletes not involved in regular resistance training	Good
Nosaka [42]	S group: 11 L group: 11	Age 20.3 ± 1.6 years Height 170.1 ± 5.7 cm Mass 61.7 ± 8.7 kg	Experimental group: S group performed eccentric exercise at a short starting length (S) first followed 4 weeks later by eccentric exercise at the long starting length Control group: L group performed eccentric exercise at a long starting length for both first (L)	Male non-athletes not involved in regular resistance training	Good
Tseng [19]	10 MVC: 13 No pre-conditioning group: 13	Experimental and no pre-conditioning groups: Age 21.0 ± 1.2 years Height 171.9 ± 5.6 cm Mass 66.4 ± 8.5 kg	Experimental group: 10 MVC Control group: No pre-conditioning group	Untrained participants who had not participated in regular resistance, aerobic or flexibility training in the previous 1 year	Good

*DW* downhill walking, *ECC* eccentric, *ECC-S* eccentric slow contraction, *ECC-F* eccentric fast contraction, *ISO* isometric, *LW* level walking, *MVC* maximal voluntary contraction, *MVC-CON* maximal voluntary concentric contraction *VO*_*2max*_ maximal oxygen consumption

### Methodological Descriptions

For the research design, 11 studies included a control group with no pre-conditioning activities, whilst eight studies incorporated groups where all participants performed pre-conditioning activities. Various types of interventions were used as pre-conditioning strategies to prevent EIMD (Table 2), with the most common being low-intensity or low-volume eccentric contractions (11 studies), followed by maximal isometric contractions (six studies), Smith machine squatting (one study), and downhill or level walking (one study). One study used both isometric and eccentric contractions as pre-conditioning, whilst one study used maximal isometric and eccentric contractions in separate groups. A variety of exercises were used to cause EIMD (Table 2), with the most common being eccentric contractions (15 studies) followed by downhill running (two studies), downhill walking (one study) and squatting (one study). The most common biomarkers for indirect muscle damage were CK (18) and myoglobin (10). One study did not measure blood biomarkers of EIMD. The level of DOMS was reported by 19 studies using visual analogue scales of 1–10, 1–100 or 1–200, with greater ratings indicating higher perceived soreness. Muscle swelling was reported using upper arm girth (eight studies), thigh girth (two studies) and ultrasound (six studies). Physical performance was measured using MVC of either the elbow flexors or knee extensors (eight studies), MVC torque of either the elbow or knee extensors (ten studies), running economy (one study) and 3-km time trial (one study). In addition, 11 studies examined ROM of the elbow joint or knee joint. Time between pre-conditioning and muscle damaging protocols varied among studies; some studies utilised more than one time period between groups. The most common durations between pre-conditioning and muscle-damaging protocols were 2 days (eight studies), 2 weeks (eight studies), 1 week (six studies) and 3 weeks (five studies).

**Table 2:** Methodological description and qualitative results of functional performance outcome measures

Study (first author)	Pre-conditioning protocol	Duration^a^	Muscle damaging protocol	Comparative group	Outcome measures
Barreto [17]	10 MVC with elbow flexed at 20°	2 days	ECC-S group performed 30 maximal eccentric contractions at 60°/seconds ECC-F group performed 90 maximal eccentric contractions at 180°/seconds	Experimental group: ISO + ECC-S and ISO + ECC-F Control group: ECC-S and ECC-F	Maximal isokinetic torque concentric torque, muscle soreness, muscle thickness
Brown [28]	10 (ECC 10), 30 (ECC 30) or 50 (ECC 50) maximal voluntary eccentric contractions of the knee extensors at velocity of 1.05 rad/s	3 weeks	50 maximal voluntary eccentric contractions of the knee extensors of the same leg at velocity of 1.05 rad/s	Experimental group: ECC 10 and ECC 30 following the second bout after the muscle-damaging protocol Control group: ECC 50 following the first bout before the muscle-damaging protocol	Maximal voluntary contraction, CK activity, 20:100 Hz stimulation force, muscle soreness
Burt [37]	Low–high group performed 5 sets of 10 squats at 80% body weight in Smith machine High-high group performed 10 sets of 10 squats at 80% body weight in Smith machine	2 weeks	10 sets of 10 squats at 80% body weight in Smith machine	Experimental group: Low–high following the second bout after the muscle-damaging protocol Control group: High-high following the first bout before the muscle-damaging protocol	Muscle soreness, plasma CK, knee extensor torque, RPE, 3-km time trial, average speed, blood lactate, ending RPE, HR, mean VO_2_
Chen [21]	5 sets of six ECC of the elbow flexors set at 10% of MVC, with dumbbells	2 days, 1 week, 2 weeks or 3 weeks	5 sets of 6 maximal eccentric contractions at angular velocity of 30°/s, 2 min between sets	Experimental group: 2-day group, 7-day group, 14-day group, 21-day group Control group: No pre-conditioning	Elbow flexor MVC, optimum angle, upper arm circumference, ROM, muscle soreness, plasma CK, Mb concentration, ultrasound echo intensity
Chen [2]	30 eccentric contractions of the elbow flexors with dumbbell set at 40%, 60%, 80% or 100% of participants MVC, repeated every 45 s	2–3 weeks	30 eccentric contractions of the elbow flexors with dumbbell set at 100% of participants MVC, repeated every 45 s	Experimental group: ECC 80%, ECC 60% and ECC 40% Control group: ECC 100% following the first bout	Optimum angle, maximal isometric strength, upper arm circumference, CK activity, Mb concentration, ROM, muscle soreness
Chen [38]	ECC groups performed 30 eccentric contractions using dumbbell set to 10%, 20% or 100% MVC. Isometric groups performed 30, 5 s maximal isometric contractions at 90° or 20° elbow flexion	3 weeks	Maximum ECC	Experimental group: Control group:	MVC torque, flexed elbow joint angle, ROM, upper arm circumference, CK activity, Mb concentration, muscle soreness
Chen [13]	6 sets of 10 eccentric contractions of knee extensors at 10% of MVC	1 week	6 sets of 10 maximal eccentric contractions of knee extensors at 30°/s	Experimental group: Control group:	MVC-CON torque, upper thigh circumference, muscle soreness, plasma CK, Mb concentration, ROM, ultrasound echo intensity
Chen [10]	2 or 10 maximal isometric contractions of the elbow flexors, elbow joint angle of 20°	2 days	5 sets of 6 maximal eccentric contractions of the elbow flexors, angular velocity 90°/s, 2 min between sets	Experimental group: Control group:	Peak torque, upper arm circumference, ROM, MVC peak torque, plasma CK, plasma Mb, ultrasound echo intensity, muscle soreness
Chen [15]	2 maximal voluntary isometric contractions of the elbow flexors, elbow angle 20°	0 days, 2 days, 4 days, 7 days	5 sets of 6 maximal voluntary eccentric contractions at angular velocity of 90°/s, 2 min between sets	Experimental group: Control group:	MVC-CON, optimum angle, upper arm circumference, muscle soreness, plasma CK, Mb concentration, flexed elbow joint angle and extended elbow joint angle, ROM, ultrasound echo intensity
Eston [3]	100 continuous maximal voluntary eccentric contractions	2 weeks	5 × 8-min bouts of downhill running at an incline of − 10% with 2 min between bouts. Speed set to elicit 80% of age-predicted heart rate	Experimental group: Control group:	Muscle soreness (mm) or CK activity, peak torque concentric (Nm), peak torque eccentric (Nm)
Howatson [39]	ECC 10 group performed 10 maximal eccentric contractions of the elbow flexors ECC 45 group 3 sets of 15 maximal eccentric contractions with 3 min between sets	2 weeks	3 sets of 15 maximal eccentric contractions of the elbow flexors at 30°/s with 3 min between sets	Experimental group: Control group:	Muscle soreness (mm), percentage change in isometric torque, percentage change in total ROM, log CK
Huang [22]	5 sets of 10 eccentric contractions at 10% MVC	2 days	5 sets of 10 eccentric contractions at 80% MVC	Experimental group: Control group:	MVC strength, muscle soreness, plasma CK, Mb concentration
Paddon-Jones [40]	LV + ECC and ECC group performed 3 maximal eccentric contractions of the elbow flexors at 0.52 and 3.14 rads/s, 3 maximal isometric contractions and 3 maximal concentric contractions at 0.52 and 3.14 rads/s	10 days and 7 days	6 sets of 6 maximal eccentric contractions at 0.52 rad/s with 60 s between sets	Experimental group: Control group:	Arm girth, elbow angle, muscle soreness, isometric torque, plasma CK, concentric torque, eccentric torque
Lavender [14]	6 sets of 5 eccentric contractions of the elbow flexors, lowering a dumbbell set at 10% of MVC	2 days	6 sets of 5 eccentric contractions of the elbow flexors, lowering a dumbbell set at 40% of MVC	Experimental group: Control group:	Maximal voluntary contraction, ROM, upper arm circumference, plasma CK, muscle soreness
Lima [20]	10 maximal voluntary isometric contractions, leg press machine at 100° of knee extension	2 days	30-min downhill run on treadmill set at − 15% incline speed set at 70% *V*O_2max_	Experimental group: Control group:	Running economy, isometric peak torque, muscle soreness, CK activity, counter-movement jump height
Lin [23]	Knee flexors 3 sets of 10, knee extensors 6 sets of 10 low intensity eccentric contractions	2 days, 1 week, 2 weeks or 3 weeks	Knee flexors 3 sets of 10, knee extensors 6 sets of 10 of maximal eccentric contractions	Experimental group: Control group:	MVC-CON torque, peak torque, ROM, plasma CK, Mb concentration, muscle soreness, ultrasound echo intensity
Maeo [7]	20-min DW (− 28% slope, 5 km/hour, 10% body mass added to a backpack)	1 week, 4 weeks	40-min DW (− 28% slope, 5 km/hour, 10% body mass added to a backpack)	Experimental group: pre 1 week, pre 4 weeks Control group: No pre-conditioning	MVC, CK, muscle soreness
Maeo [16]	Downhill walking at a − 28% incline at 5 km/hour with 10% body mass added for 5 min, level walking at 0% incline at 5 km/h with 10% body mass added for 5 min	1 week	Downhill walking at a − 28% incline at 5 km/h with 10% body mass added for 40 min	Experimental group: Control group:	Maximal voluntary contraction torque, plasma CK, muscle soreness
Miyama [41]	5 sets of 10 DJs	2 weeks	5 sets of 10 DJs	Experimental group: Performed 5 sets of 10 DJs in the second bout Control: Performed 5 sets of 10 DJs in the first bout	CMJ height, SJ height, peak vertical GRF, ground contact time, blood lactate, heart rate, maximal isometric torque, muscle soreness, CK
Nosaka [8]	12 maximal eccentric actions of the elbow extensors with each arm	2 weeks	100 repetitions of isokinetic concentric contractions of the elbow extensors at an angular velocity of 1.05 rad/s followed by 12 maximal eccentric actions of the elbow extensors with each arm	Experimental group: CON-ECC Control group: ECC	Muscle soreness, isometric force, ROM, CK
Nosaka [18]	2, 6 or 24 maximal eccentric contractions of the elbow flexors, performed every 15 s	2 weeks	24 eccentric contractions of the elbow flexors every 15 s	Experimental group: Control group:	Maximal isometric force, upper arm circumference, ROM, relaxed elbow joint angle, flexed elbow joint angle, muscle soreness, plasma CK, Mb concentration
Nosaka [42]	Eccentric action from more elbow flexed position (50°) and ended at 100°, with 2 s duration of eccentric action after a 1-s maximal isometric contraction	4 weeks	Subject’s elbow joint was forcibly extended post 1 s of maximal isometric contraction from a flexed (130°) to an extended (180°) position in 2 s	Experimental group: S group performed eccentric exercise at a short starting length (S) first followed 4 weeks later by eccentric exercise at the long starting length Control group: L group performed eccentric exercise at a long starting length for both first (L) and second bouts	Muscle soreness, isometric force, ROM, CK, echo intensity
Tseng [19]	6 sets of 10 maximal isometric contractions of the knee extensors at 90° knee joint angle, 2 min between sets	2 weeks	6 sets of 10 maximal eccentric contractions of the knee extensors, angular velocity of 30°/s. 2 min between sets	Experimental group: Control group:	MVC-CON, thigh girth, muscle soreness, plasma CK, Mb concentration, ROM, ultrasound echo intensity

*CK* creatine kinase, *ECC* eccentric, *ECC-F* fast velocity eccentric contractions, *ECC-S* slow-velocity eccentric contractions, *HR* heart rate, *Hz* hertz, *LV* low volume, *Mb* myoglobin, *MVC* maximal voluntary contraction, *ROM* range of motion, *RPE* rating of perceived exertion, *VO*_*2*_ oxygen consumption, *VO*_*2max*_ maximal oxygen consumption

^a^Duration between the pre-conditioning activity and muscle damaging protocol

### Methodological Quality and Risk of Bias

The Kmet values for included studies ranged from good to excellent (Table 3). All included studies met the following criteria: eligibility criteria; similar measures at baseline and days 1–4; participants shared a similar training background; use of a pre-conditioning protocol; EIMD protocol performed more than 24 h after pre-conditioning; reported two or more outcome measures associated with EIMD (e.g. CK, myoglobin, ROM, MVC, DOMS or muscle girth); and results reported with measures of central tendency and dispersion. According to Egger’s test, all parameters at each time point were statistically significant (*p* < 0.05), indicating a publication bias.

**Table 3:** Quality ratings of studies

Study (first author)	1	2	3	4	5	6	7	8	9	10	11	12	13	14	Total scores^a^
Barreto [17]	2	2	1	2	2	1	1	2	2	2	2	2	2	2	25a
Brown [28]	2	2	1	1	1	0	1	2	2	2	2	2	2	1	21b
Burt [37]	2	2	1	2	1	2	1	2	1	2	2	2	2	2	24a
Chen [21]	2	2	2	2	2	0	1	2	2	2	2	2	2	1	24a
Chen [2]	1	2	1	1	0	0	2	2	2	2	2	2	2	1	20b
Chen [38]	2	2	1	2	1	0	1	2	2	2	2	2	2	1	22b
Chen [13]	2	2	1	1	1	2	2	2	2	2	2	2	2	1	24a
Chen [10]	2	2	1	2	2	2	2	2	2	2	2	2	2	2	27a
Chen [15]	2	2	1	2	2	1	2	2	2	2	2	2	2	2	26a
Eston [3]	1	2	1	1	1	2	1	2	1	2	2	1	2	2	21b
Howatson [39]	1	2	1	0	1	1	1	2	1	2	2	1	2	2	19b
Huang [22]	2	2	1	1	1	0	1	2	2	2	2	2	2	2	22b
Paddon-Jones [40]	2	2	1	1	2	0	2	2	1	2	2	2	2	2	23b
Lavender [14]	2	2	1	2	1	0	1	2	2	2	2	2	2	2	23b
Liman [20]	2	2	1	2	1	0	1	2	1	2	2	2	2	2	20b
Lin [23]	1	2	1	2	1	0	1	2	2	2	2	2	2	1	21b
Maeo [7]	2	2	1	2	2	2	1	2	1	2	2	2	2	2	25a
Maeo [16]	2	2	1	2	1	0	1	2	2	2	2	2	2	1	22b
Miyama [41]	2	2	1	1	2	0	1	2	1	2	2	2	2	2	22b
Nosaka [8]	2	2	1	1	0	0	1	2	1	2	2	2	2	1	19b
Nosaka [18]	2	2	1	1	1	1	1	2	1	2	1	2	2	1	20b
Nosaka [42]	2	2	1	1	2	0	1	2	1	2	2	2	2	2	22b
Tseng [19]	2	2	1	2	2	1	1	2	2	2	2	2	2	1	24a

Item description: 1. Objective; 2. Design/study question; 3. Method of subject selection; 4. Subject characteristics; 5. Randomisation; 6. Baseline or pre-test measures included eccentric or isometric contractions; 7. Dependent measures taken at the same time of day; 8. Outcome measures; 9. Sample size; 10. Statistics and analyses; 11. Estimate of variance; 12. Dependent variables recorded at appropriate intervals; 13. Results reported; 14. Conclusion stated

^a^Study quality rated as a = excellent > 23, b = good > 18, c = fair > 14 or d = poor < 14

### Quantitative Analysis

The pre-conditioning groups demonstrated significantly lower CK levels than EIMD-only groups at 24 h (SMD =  − 1.42; Z = 8.00; *p* = 0.00001), 48 h (SMD =  − 2.21; Z = 9.50; *p* = 0.00001), 72 h (SMD =  − 2.21; Z = 9.50; *p* = 0.00001) and 96 h post-exercise (SMD =  − 3.22; Z = 9.57; *p* = 0.00001). In addition, substantial inter-study heterogeneity was identified for CK at 24 h (*I*^2^ = 82%; $\chi^{2}$ = 219.0), 48 h (*I*^2^ = 88%; $\chi^{2}$ = 345.8), 72 h (*I*^2^ = 87%; $\chi^{2}$ = 283.0) and 96 h (*I*^2^ = 91%; $\chi^{2}$ = 418.5) [Electronic Supplementary Material (ESM)]. Similarly, DOMS was significantly lower in the pre-conditioning groups than EIMD only groups at 24 h (SMD =  − 1.94; *Z* = 8.27; *p* = 0.00001), 48 h (SMD =  − 2.52; *Z* = 10.50; *p* = 0.00001), 72 h (SMD =  − 2.70; *Z* = 10.61; *p* = 0.00001) and 96 h post-exercise (SMD =  − 2.21; *Z* = 9.99; *p* = 0.00001), with substantial inter-study heterogeneity at 24 h (*I*^2^ = 89%; $\chi^{2}$ = 416.7), 48 h (*I*^2^ = 89%; $\chi^{2}$ = 405.9), 72 h (*I*^2^ = 89%; $\chi^{2}$ = 392.0) and 96 h (*I*^2^ = 89%; $\chi^{2}$ = 379.7) [ESM]. The pre-conditioning groups maintained MVC and returned to pre EIMD levels sooner than the EIMD-only groups at 24 h (SMD = 1.47; *Z* = 6.85; *p* = 0.00001), 48 h (SMD = 1.63; *Z* = 7.26; *p* = 0.00001), 72 h (SMD = 1.99; *Z* = 7.48; *p* = 0.00001) and 96 h post-exercise (SMD = 2.11; *Z* = 6.36; *p* = 0.00001), with substantial inter-study heterogeneity identified for MVC at 24 h (*I*^2^ = 82%; $\chi^{2}$ = 147.6), 48 h (*I*^2^ = 81%; $\chi^{2}$ = 148.3), 72 h (*I*^2^ = 86%; $\chi^{2}$ = 197.4) and 96 h (*I*^2^ = 89%; $\chi^{2}$ = 185.9) [ESM]. Finally, the pre-conditioning groups better maintained ROM after EIMD compared with EIMD-only groups at 24 h (SMD = 1.43; *Z* = 5.89; *p* = 0.00001), 48 h (SMD = 2.05; *Z* = 7.50; *p* = 0.00001), 72 h (SMD = 2.52; *Z* = 7.79; *p* = 0.00001) and 96 h post-exercise (SMD = 2.43; *Z* = 7.65; *p* = 0.00001), with substantial inter-study heterogeneity present at 24 h (*I*^2^ = 86%; $\chi^{2}$ = 201.4), 48 h (*I*^2^ = 88%; $\chi^{2}$ = 236.4), 72 h (*I*^2^ = 90%; $\chi^{2}$ = 272.4) and 96 h (*I*^2^ = 90%; $\chi^{2}$ = 290.1) [ESM].

### Qualitative Analysis

#### Level of Protection from EIMD from Various Pre-Conditioning Protocols

When examining individual studies that compared various pre-conditioning protocols, the contraction type, intensity, volume, and joint angle of pre-conditioning activities and the duration between pre-conditioning activities and the muscle-damaging protocol appeared to influence the magnitude of protection from EIMD following subsequent muscle-damaging exercises. For example, with respect to the duration between pre-conditioning activities and the EIMD protocol, significantly lower EIMD was reported when the pre-conditioning activities (30 eccentric contractions at 10% MVC of elbow [21] and knee [23] flexors, two repetitions of MVC of elbow flexors [15]) were performed 2–4 days before the muscle-damaging exercises when compared with the pre-conditioning activities performed 1–3 weeks before the muscle-damaging exercises [21, 23] and immediately before the muscle-damaging protocol [15]. With respect to the volume of pre-conditioning activities, a greater volume of the pre-conditioning activity performed before the muscle-damaging exercises also exhibited lower levels of EIMD, such as the comparison between 10 repetitions of MVC to two repetitions of MVC held for 3 s during each MVC [10] and six eccentric contractions to two eccentric contractions [18]. However, results from one study showed no differences in the level of EIMD between pre-conditioning activities of 10 and 30 eccentric contractions [28]. Pre-conditioning activities performed with higher intensity showed lower levels of EIMD following muscle-damaging exercises, such as the comparison between 30 eccentric contractions performed at 80% of MVC to 40% and 60% of MVC, although no differences were found between 40 and 60% of MVC [2]. Furthermore, the level of EIMD was lower with pre-conditioning activities of 30 eccentric contractions performed at 20% of MVC than with 10% of MVC before the muscle-damaging exercises [38]. Interestingly, when comparing these two studies based on our forest plots, the SMD values were relatively similar for 30 eccentric contractions performed at 10–20% of MVC [38] and 40–60% of MVC [2] for CK, DOMS and MVC measures (ESM), although lower EIMD levels and a faster recovery of MVC were found with 30 eccentric contractions performed at 80% of MVC [2]. Downhill walking for 5 min also exhibited lower levels of EIMD as a pre-conditioning activity when compared with flat walking for 5 min [16], further supporting greater protection from EIMD with a higher intensity of pre-conditioning exercises. When comparing contraction types, eccentric contractions exhibited a greater protection from EIMD than isometric contractions [38]. Finally, pre-conditioning activities consisting of 30 repetitions of MVC held for 3 s during each contraction performed at an elbow angle of 20° (lengthened elbow flexors) showed greater protection from EIMD than at an elbow angle of 90° (shortened elbow flexors).

#### Level of EIMD After Various Pre-Conditioning Activities

When examining the acute responses to the pre-conditioning exercises, no significant differences in EIMD markers (i.e. CK, DOMS and MVC) were reported after 5 min of flat and downhill walking [16], 30 sub-maximal eccentric contractions of knee flexors and 60 eccentric contractions of knee extensors performed at 10% of MVC [23], 30 sub-maximal eccentric contractions at 10% MVC of elbow flexors [21] and two repetitions of MVC performed at 20° of an elbow flexion angle held for 3 s during each contraction [10, 15]. However, a significant increase in DOMS and a reduction in MVC were reported after 10 and 30 maximal eccentric contractions for at least 72 h post-exercise [28]. In addition, significant increases in CK and DOMS and a loss in MVC were reported after 30 sub-maximal eccentric contractions performed at 40%, 60% and 80% of MVC for at least 48-h post-exercise [2]. Furthermore, DOMS was significantly increased for 48 h and MVC was impaired for at least 24 h after 30 sub-maximal eccentric contractions at 10% and 20% of MVC, and 30 repetitions of MVC at 20° of an elbow flexion angle [38]. However, in the same study [38], DOMS was significantly increased, whilst MVC was decreased for only 24 h after 30 repetitions of MVC at 90° of an elbow flexion angle. Differences between baseline and post-exercise were not reported after the pre-conditioning activity for EIMD in one study [18], although CK and DOMS were notably increased for at least 48 h after 2 and 6 repetitions of maximal eccentric contractions, with 6 repetitions of maximal eccentric contractions exhibiting significantly greater EIMD than 2 maximal eccentric contractions.

### Certainty of Evidence

The certainty of evidence for all outcomes (Table 4) was very low based primarily on the limited sample size for comparison (*n* < 800), publication bias and high inter-study heterogeneity (I^2^ > 75%).

**Table 4:** Certainty of evidence for meta-analysed outcome

Outcome	No. of participants	Certainty of evidence
CK	730	24 h: very low^a,b,c^ 48 h: very low^a,b,c^ 72 h: very low^a,b,c^ 96 h: very low^a,b,c^
DOMS	794	24 h: very low^a,b,c^ 48 h: very low^a,b,c^ 72 h: very low^a,b,c^ 96 h: very low^a,b,c^
MVC	573	24 h: very low^a,b,c^ 48 h: low^a,b^ 72 h: very low^a,b,c^ 96 h: very low^a,b,c^
ROM	444	24 h: low^a,b^ 48 h: very low^a,b,c^ 72 h: very low^a,b,c^ 96 h: very low^a,b,c^

*CK* creatine kinase, *DOMS* delayed-onset muscle soreness, *MVC* maximum voluntary contraction, *ROM* range of motion

^a^Downgraded by one level because of < 800 participants

^b^Downgraded by one level if publication bias was suspected

^c^Downgraded by one level if the inter-study heterogeneity (I^2^) was high (> 75%)

## Discussion

The current systematic review and meta-analysis examined the extent to which pre-conditioning interventions attenuate EIMD. Applying stringent inclusion criteria, 19 articles were included in the meta-analysis. Based on meta-analytical data, pre-conditioning interventions attenuated the level of EIMD (i.e. CK, DOMS, ROM and MVC) when performed a minimum of 24 h prior to a bout of strenuous exercise, compared with groups without pre-conditioning activities. In addition, the participants recovered from EIMD more quickly when exposed to pre-conditioning activities, with notably greater SMD values from 24 h post-exercise to 72 h post exercise. Furthermore, the pre-conditioning activities appeared to be most effective with the following: when implemented 2–4 days prior to the EIMD protocols; when pre-conditioning activities were performed with greater volume (10–30 eccentric contractions) compared to lower volume (2–6 eccentric contractions); when pre-conditioning activities were performed at higher intensities (20–80% of MVC) as opposed to lower intensities (10% MVC); when pre-conditioning activities were performed with more lengthened muscles; and eccentric contractions exhibited greater protection than isometric contractions. Whilst several of the pre-conditioning activities employed in these studies also increased the level of EIMD (elevated CK and DOMS and decreased MVC), the level of EIMD was notably lower than in the control group after the EIMD protocol. Thus, implementing pre-conditioning activities as a priming method prior to the initial exposure of muscle-damaging exercises alleviates symptoms of EIMD, and may accelerate recovery.

According to the traditional concept of RBE, a high level of EIMD is expected following the first bout of muscle-damaging exercises to minimise the level of EIMD following the second bout of similar, or identical muscle-damaging exercises [6, 11]. However, studies in this review achieved a similar prophylactic response to the RBE using pre-conditioning, bypassing the need for an initial high level of EIMD response. Pre-conditioning activities may provide an alternative with the benefits of the RBE that can be tailored for a specific outcome. A potent RBE brought about by traditional means of EIMD such as heavy strength training may attenuate subsequent muscle damage for months [2, 13]. However, the duration of protection from the pre-conditioning intervention appears to be specific to the type of intervention chosen, with low-volume maximal isometric pre-conditioning (IPC) utilising between two and ten isometric contractions attenuating muscle damage for only a few days (2–4 days) [10, 15, 17], while higher volume IPC appears to maintain attenuation potential for several weeks [13, 19].

The use of eccentric pre-conditioning has also been shown to maintain protective capacity against EIMD for weeks to months using low-volume maximal eccentric contractions to attenuate a second bout of high-volume ECC of the same exercise [18, 28]. Similar protective effects have been observed using sub-maximal intensity (10%) EPC to attenuate EIMD caused by a second damaging bout of high-volume and high-intensity ECC of the same exercise [13, 22]. The data in this review show that IPC interventions using between 2 and 60 isometric contractions provide adequate attenuation from CK, DOMS, ROM and MVC [10, 15, 19]. Similarly, EPC between 2 and 60 ECC contractions or sub-maximal ECC (10% MVC) also successfully attenuates EIMD [2, 10, 13, 22]. Both IPC and EPC can be applied in a range of scenarios and tailored to the specific needs of an individual’s programme where EIMD may not be desirable.

Several mechanisms have been proposed to explain how pre-conditioning activities attenuate muscle damage in the absence of any noticeable stimulus. Type IV collagen within the extracellular matrix (ECM) may interact with isometric and low-intensity ECC, allowing for a slow contraction of the type IV fibres. The alignment of ECM in this way may improve the transduction of force through the musculoskeletal system [43]. The slow contraction of the ECM may explain why EIMD will occur when exercise is completed immediately after pre-conditioning, although attenuated 24 h later. Furthermore, eccentric contractions as pre-conditioning activities have been suggested to upregulate the cytoskeletal proteins, which may strengthen the cytoskeletal protein network by stabilising the sarcomeres during eccentric contractions in subsequent muscle-damaging bouts [44]. It has also been suggested that pre-conditioning exercises may upregulate anti-oxidant enzymes whilst concomitantly reducing inflammatory markers, which may attenuate the secondary muscle damage response following subsequent muscle-damaging bouts [45, 46]. The duration of protection, particularly from maximal isometric contractions, is short lived, lasting only several days. It is therefore possible that non-damaging contractions may prevent EIMD by facilitating greater force output and muscle fibre recruitment as a result of familiarisation. In addition, muscle length appears to be an important factor in the success of a pre-conditioning strategy, as force production is greatly affected by muscle length afforded by the angle of a joint [47–49]. In the study by Chen et al. [38], 90° of elbow extension produced a less potent result than 20° of elbow extension during isometric contractions, highlighting the importance of muscle length when attempting pre-conditioning for EIMD. Ofori et al. [48] observed that muscle fibre recruitment and force production were affected by fibre length and ROM of the muscle being used, with force production greatest when muscle fibre length was long. This allowed for greater fibre recruitment while taking advantage of the mechanical and neural properties afforded by fibre elongation. The current meta-analysis exhibited significantly lower levels of EIMD for the pre-conditioning group from various interventions, suggesting that non-damaging protection against EIMD from subsequent strenuous training sessions is achievable using a variety of pre-conditioning strategies. However, the mechanisms offering protection are still debatable, and require more exploration beyond the scope of this review.

Several studies included in this review used sub-maximal isometric or concentric MVCs during familiarisation and baseline testing to reduce interference with the chosen pre-conditioning strategy [10, 13, 37]. While it is generally accepted that concentric contractions do not confer muscle damage at low volumes [1, 50], it remains unclear as to what extent concentric contractions affect the outcomes associated with EIMD. Similarly, the use of sub-maximal isometric contractions during familiarisation may have conferred some degree of pre-conditioning. Whilst this was not evident in the data within this review, the exploration of isometric thresholds that exhibit protection from EIMD may be valuable in broadening the application of pre-conditioning. Research implementing MVC and ECC as outcome measures should also consider how the implementation of MVC at baseline will impact their outcomes. The studies included in this review highlight that pre-conditioning can affect MVC, ROM and other measures of performance for up to 3 weeks, while some studies have shown that a single bout of ECC can have a RBE lasting several months [18]. This review corroborates suggestions made by Chen et al. [15] and Nosaka et al. [18] that studies examining the level of EIMD of an intervention should implement a washout period, allowing adequate time for interference from pre-testing and/or baseline measures to dissipate.

It is unclear exactly which pre-conditioning strategies would be effective for use prior to athletic performance. However, based on data collected from this review, it is possible to speculate that pre-conditioning may attenuate EIMD resulting from strenuous training. Similarly, pre-conditioning may improve concurrent training outcomes. Pre-conditioning could be implemented into periodisation plans to reduce EIMD between training sessions, improving the quality of cardiovascular training that occurs post-resistance training, particularly for endurance athletes for whom resistance training is less frequent or when commencing resistance training after a break or as part of a new training regime. This would ultimately lead to greater training quality, allowing full ROM and force production while reducing the injury risk during training for endurance athletes [16, 20].

The complex nature of sporting competition makes speculation regarding the effectiveness and appropriate application of pre-conditioning for athletes challenging. Further evaluation is therefore required to explore the use and effectiveness of pre-conditioning in sport. Many studies in this review utilised mono-articular pre-conditioning interventions. Mono-articular movement does not replicate the complexity of most training exercises, utilising only a single joint and only the muscles that cross that joint. Multi-articular pre-conditioning offers a greater opportunity for pre-conditioning to transfer to the real world, using multiple joints and muscles working at varying angles across an entire limb [19, 20, 22]. Lima et al. [20] implemented multi-articular isometric contractions using a leg press machine followed by a bout of downhill running. The EIMD induced by downhill running was attenuated by the isometric leg press as measured by CK, MVC and muscle soreness; however, running kinematics and metabolism were not significantly different between groups. This suggests the prophylactic effects of pre-conditioning can be transferred across different exercises if the same muscle groups are targeted. No studies included in this review implemented multiple exercises targeting the same muscle group in their second bout designed to cause EIMD. Traditional resistance training sessions typically consist of multiple exercises with a focus on developing a particular athletic attribute (power, strength, strength endurance). Huang et al. [22] did use multiple exercises to assess if EIMD could be attenuated by more than one muscular group; however, each exercise was preceded by a pre-conditioning activity specific to that exercise (e.g. 5 sets of 10 repetitions at 10% ECC on the lat pull down machine, followed 48 h later by 5 sets of 10 repetitions at 80% ECC on the lat pull down machine). It therefore remains unclear to what extent EIMD will be attenuated by a multi-articular pre-conditioning activity following a bout of traditional resistance exercises that are performed with multi-articular activities.

During methodological quality assessment of studies in the current systematic review, several areas were identified as generally scoring low using the Kmet criteria. These include:

- Method of subject selection: the majority of studies scored 1 in this area because of the lack of specificity regarding subjects' training history. Subjects were generally described as healthy, untrained or non-resistance trained in some capacity; however, these criteria were not always well defined and exclusion criteria were not always clear. Greater specificity regarding subject inclusion criteria is necessary to ensure that a variation in participants' training volume or daily activities does affect the degree of protection afforded by pre-conditioning strategies.
- If baseline or pre-test measures included eccentric or isometric contractions, was a washout period observed prior to treatment commencing: many studies scored a 0 or 1 in this area, as there was either no washout period observed or it was unclear when pre-testing took place prior to intervention. If a washout period is not considered before the intervention, any response will be affected by pre-test measures, thus confounding the EIMD response during the intervention. Future studies should implement a washout period prior to the intervention to allow for any pre-conditioning that occurs because of pre-testing to dissipate.
- Dependent variables taken at the same time of day or within a specified window: many studies in this review scored a 1 on this item. The outcome measures of DOMS, MVC and CK are affected by the time of day and the duration between sessions, and as such, the specificity regarding subject attendance time is important. Many studies in this review included time points in tables and figures but did not specify if these measures were taken at approximately 24 h or within a window of time after 24 h. It is important that future studies are specific when participants attended consecutive sessions and within what window of time, as this may affect outcome measures.

It is unclear if pre-conditioning attenuates more than one bout of intense exercise performed over multiple days, and thus future research could examine the effectiveness of pre-conditioning exercises across multiple muscle-damaging bouts. Furthermore, future research should consider how isometric pre-conditioning can be implemented practically for athletes involved in concurrent training; identify at what thresholds isometric pre-conditioning provides attenuation; and determine whether isometric pre-conditioning can be used to attenuate muscle damage during multiple compound exercises such as those performed in a typical resistance training session. Such information would be useful for athletes during a high-load training phase or during intense competition phases. Finally, the type of pre-conditioning protocols and muscle-damaging protocols varied between studies, which may have affected the severity of EIMD. Eccentric exercise is an effective pre-conditioning strategy for attenuating muscle damage; however, this type of contraction produces some level of muscle damage as evidenced by previous studies [2, 18, 28].

The variation in pre-conditioning strategies included was a limitation of the current review, particularly the variation in pre-conditioning intensity amongst eccentric-based interventions, which possibly contributed to high inter-study heterogeneity in most cases (*I*^2^ > 75%). A further limitation of the review was that studies were combined with varying timing between pre-conditioning and the second bout of damaging exercise, different muscle groups (upper body vs lower body) and contraction types (isometric vs eccentric) into our meta-analyses. Although the current meta-analysis indicated that pre-conditioning activities reduce the level of EIMD following subsequent EIMD bouts, it is important to note that publication bias was present in all outcome measures, and the certainty of evidence was low. Finally, whilst every effort was made to contact the authors of each paper, most data were extracted from figures using data extracting software (ImageJ), which may result in some variation from the original results of each paper.

## Conclusions

The current systematic review identified that pre-conditioning activities are effective in attenuating EIMD measures, CK, muscle soreness, ROM and maximal voluntary force production. These findings suggest the use of pre-conditioning activities prior to a strenuous bout of exercise does provide a significant protective effect when performed a minimum of 24 h prior to the damaging stimulus. Furthermore, the greater improvement in MVC suggests that the pre-conditioning activities may accelerate the recovery of muscle contractile properties following strenuous exercises. However, it is unclear if pre-conditioning would prevent EIMD from more complex exercises or to what extent EIMD prevention carries over into other modes of exercise.

## Supplementary Information

Below is the link to the electronic supplementary material.

Supplementary file 1. Forest plots effect of pre-conditioning on creatine kinases release 24 hours post damaging stimulus

Supplementary file 2. Forest plots effect of pre-conditioning on creatine kinases release 48 hours post damaging stimulus

Supplementary file 3. Forest plots effect of pre-conditioning on creatine kinases release 72 hours post damaging stimulus

Supplementary file 4. Forest plots of pre-conditioning on creatine kinases release 96 hours post damaging stimulus

Supplementary file 5. Forest plots showing effect of pre-conditioning on delayed onset muscle soreness 24 hours post damaging stimulus

Supplementary file 6. Forest plots showing effect of pre-conditioning on delayed onset muscle soreness 48 hours post damaging stimulus

Supplementary file 7. Forest plots showing effect of pre-conditioning on delayed onset muscle soreness 72 hours post damaging stimulus

Supplementary file 8. Forest plots showing effect of pre-conditioning on delayed onset muscle soreness 96 hours post damaging stimulus

Supplementary file 9. Forest plots showing effect of pre-conditioning on maximal voluntary contractions 24 hours post damaging stimulus

Supplementary file 10. Forest plots showing effect of pre-conditioning on maximal voluntary contractions 48 hours post damaging stimulus

Supplementary file 11. Forest plots showing effect of pre-conditioning on maximal voluntary contractions 72 hours post damaging stimulus

Supplementary file 12. Forest plots showing effect of pre-conditioning on maximal voluntary contractions 96 hours post damaging stimulus

Supplementary file 13. Forest plots showing effect of pre-conditioning on range of motion 24 hours post damaging stimulus

Supplementary file 14. Forest plots showing effect of pre-conditioning on range of motion 48 hours post damaging stimulu

Supplementary file 15. Forest plots showing effect of pre-conditioning on range of motion 72 hours post damaging stimulus

Supplementary file 16. Forest plots showing effect of pre-conditioning on range of motion 96 hours post damaging stimulus

## Declarations

### Availability of Data and Material

Data will be provided upon request.
